# Artificial Intelligence-Guided Cosolvent Design for High-Performance Perovskite/Silicon Tandem Solar Cells

**DOI:** 10.1007/s40820-026-02291-9

**Published:** 2026-07-21

**Authors:** Lu Liu, Xinying Cai, Bita Farhadi, Xinrui Dong, Kai Wang, Yufei Shao, Shulin Wang, Jiaxue You, Wanyi Li, Hao-Chung Kuo, Hanying Wang, Dong Yang, Alex K.-Y. Jen, Shengzhong Frank Liu

**Affiliations:** 1 State Key Laboratory of Photoelectric Conversion and Utilization of Solar Energy, Center of Materials Science and Optoelectronics Engineering, Dalian Institute of Chemical Physics, Chinese Academy of Sciences, Dalian, 116023 People’s Republic of China; 2 Department of Materials Science and Engineering, City University of Hong Kong, Kowloon, 999077 Hong Kong People’s Republic of China; 3 EIT Data Science and Communication College, Zhejiang Yuexiu University, Shaoxing, 312030 People’s Republic of China; 4 Semiconductor Research Center, Hon Hai Research Institute, Taipei, Taiwan People’s Republic of China; 5 School of New Energy, Yulin University, Yulin, 719000 People’s Republic of China

**Keywords:** Machine learning, Green solvent, Wide-bandgap perovskite, Tandem solar cells, High crystallinity

## Abstract

Realizing high-performance perovskite/silicon tandem solar cells requires precise control of wide-bandgap perovskite crystallization. Solvent engineering is the most direct lever for this task; yet, its intricate, multi-variable mechanisms defy intuition-driven design. Herein, we overcome this bottleneck by pioneering a retrieval-augmented large language model to screen > 8000 solvents, identifying *γ*-valerolactone (GVL) as a non-toxic, high-performance cosolvent. It is found that the GVL strongly coordinates FA^+^, thus precisely modulating crystallization kinetics, retarding nucleation, and promoting oriented, micrometer-scale grain growth. The resulting films exhibit not only superior crystallinity, reduced non-radiative recombination, but also improved scalability to large area and the tolerance to increased film thickness. Consequently, both the single-junction and tandem devices achieve efficiencies of 23.3% and 32.5%, respectively, along with excellent stability under moisture and illumination. This study establishes the first artificial intelligence (AI)-guided cosolvent strategy for 1-μm-thick perovskite layers in perovskite/silicon tandem architectures, underscoring the transformative role of generative AI in advancing high-performance photovoltaics.

**Supplementary Information:**

The online version contains supplementary material available at 10.1007/s40820-026-02291-9.

## Highlights

- A retrieval‑augmented large language model screens >8000 solvents and identifies γ‑valerolactone (GVL) as a non‑toxic, high‑performance cosolvent for wide‑bandgap perovskites, establishing the first AI‑guided cosolvent strategy for perovskite/silicon tandem solar cells.
- GVL strongly coordinates FA^+^, delaying nucleation, promoting oriented micrometre‑scale grain growth, and enabling uniform ~1‑μm‑thick perovskite films on textured silicon with suppressed non‑radiative recombination and phase segregation.
- GVL-enabled devices achieve 23.3% (single-junction) and 32.5% (perovskite/silicon tandem) efficiencies, with excellent stability: T_80_ >4000 h (storage) and >1000 h (operational) for single-junction cells, demonstrating a scalable and sustainable solvent engineering route.

**Supplementary Information:**

The online version contains supplementary material available at 10.1007/s40820-026-02291-9.

## Graphical abstract

**Figure:**
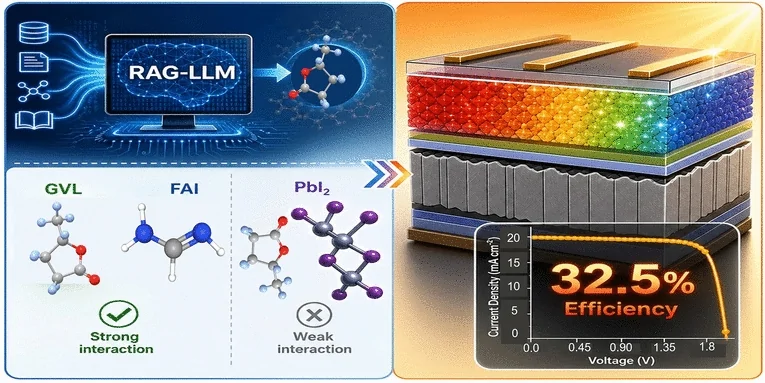

## Introduction

The monolithic integration of perovskite solar cells (PSCs) with crystalline silicon in a tandem architecture offers a promising pathway to surpass the Shockley–Queisser efficiency limit of single-junction photovoltaics [1–4]. To date, the 1-cm^2^ tandem solar cells (TSCs) have achieved a certified power conversion efficiency (PCE) exceeding 34% [5]. Achieving improvements in efficiency and operational stability critically depends on enhancing the quality of the wide-bandgap (WBG) perovskite absorbers in top sub-cells [6, 7]. However, the inherently low formation enthalpy of perovskite lattices poses considerable challenges for controlled crystallization, often resulting in point defects [8, 9], phase impurities [10–12], and spatial inhomogeneity [13, 14]. Also, the mixed halide perovskites are subject to the competition in the crystallization of different structural and compositional phases [15–17]. These imperfections are strongly linked to ion migration, phase segregation, and device degradation under operating conditions. Therefore, precise control over the crystallization dynamics of WBG perovskites is always essential to unlock their full potential in achieving high efficiency and long-term stability in tandem devices.

It is the solvents that provide the medium for dissolving solutes and play a decisive role in governing the thermodynamics and kinetics of crystallization, thereby making solvent engineering one of the most direct strategies for optimizing perovskite film quality. Till now, building upon the conventional solvent system composed of dimethylformamide (DMF) and dimethyl sulfoxide (DMSO), more than 20 alternative solvents, including amines, amides, ionic liquids, alcohols, ethers, carboxylic acid esters, and phosphates, have been explored for typical bandgap (TBG) perovskite precursors [18]. It is summarized that the interactions between solvents and solutes (e.g., coordination strength and hydrogen bonding), as well as the interactions among mixed solvents [19, 20], together with solvent intrinsic properties (such as boiling point, viscosity, vapor pressure, and polarity) [21], collectively influence precursor stability and microstructure [22], intermediate phase transition kinetics [23], and the crystallization behavior, anisotropy, and uniformity of the resulting films [24, 25]. Consequently, achieving targeted and precise control over solvent composition to optimize the crystal quality remains a significant challenge [23, 26].

Compared to TBG perovskites, wide-bandgap (WBG) perovskite precursors incorporating bromide and cesium salts exhibit stronger ionic bonding, which substantially reduces their solubility and poses significant challenges in developing suitable solvent systems. Moreover, when depositing perovskite layers onto nanotextured silicon substrates for tandem integration, achieving conformal coverage over pyramidal features requires increasing the film thickness to the micrometer scale. This, in turn, necessitates highly concentrated precursor solutions, underscoring the demand for solvents with superior dissolving capability. It is worth noting that solvent systems optimized for TBG perovskites often fail to deliver sufficient solubility for WBG counterparts [27, 28]. Although alternative solvent systems such as DMPU/DMF/DMSO (DMPU is N,N´-dimethylpropylene urea) and DMSO/acetonitrile/ethanol have been investigated for WBG perovskites [29, 30], their practical use has been mostly confined to single-junction or all-perovskite tandem devices featuring relatively thin WBG layers. Therefore, the development of innovative high-solubility solvent systems tailored for WBG perovskite materials is urgently required to precisely control crystallization dynamics and support the fabrication of high-quality, micrometer-thick films. Moreover, deliberate solvent design can also reduce dependence on hazardous solvents such as DMF, thereby enhancing the environmental sustainability of perovskite fabrication [27, 28, 31, 32].

Machine learning (ML) is a powerful data-driven approach for uncovering complex structure–property–performance relationships and has been increasingly used in PSC research to accelerate materials discovery and device optimization, including the screening of perovskite compositions, molecular additives, and interfacial passivation strategies [33–35]. The emergence of large language models (LLMs), coupled with their strong literature comprehension and data processing abilities, has accelerated the convergence of artificial intelligence and materials chemistry. Leveraging LLMs to design solvent engineering tailored for WBG perovskites is therefore both practical and efficient. Herein, we applied an ML-based screening of a large solvent dataset comprising over 8,000 of candidates, identifying four environment-friendly solvents with potential to partially replace DMF. Guided by solubility experiments, *γ*-valerolactone (GVL) emerged as a preferred cosolvent for WBG perovskites [36–38]. We then systematically investigated the interactions between GVL and perovskite precursors, its effects on solution microstructure, and its role in regulating crystallization dynamics. The results show that GVL effectively improves crystal quality and enables the formation of uniform perovskite films, with particular advantages in producing micrometer-thick layers. Notably, this work presents the first co-solvent system enabling the fabrication of micron-thick WBG perovskite films for tandem applications. Furthermore, GVL incorporation imparts perovskite films with suppressed phase segregation, extended carrier lifetimes, and reduced non-radiative recombination losses. As a result, both the optimized single-junction and tandem devices demonstrated excellent PCEs, coupled with remarkable stability under a variety of aging conditions.

## Experimental Section

### Materials

Tetrahydrofuran (THF, 99%), 2-methyltetrahydrofuran (2-MeTHF, 99%), and 1-pentanol (1-P, 99%) were purchased from Adamas. γ-valerolactone (GVL, 98%) and n-butyl acetate (BAC, 99%) were purchased from Aladdin Biochemical Technology Co., Ltd. N,N-dimethylformamide (DMF, 99.8%), and dimethyl sulfoxide (DMSO, 99.9%) were purchased from Sigma-Aldrich. Isopropanol (IPA, 99.5%) was purchased from J&K. Lead iodide (PbI_2_, 99.999%), lead bromide (PbBr_2_, 99.99%), cesium iodide (CsI, 99.99%), and hole blocking material 2,9-dimethyl-4,7-diphenyl-1,10-phenanthroline (BCP, 99.5%) were purchased from Xi'an Yuri Solar Co., Ltd. Formamidinium iodide (H_2_N = CHNH_2_I; FAI), [6, 6]-phenyl-C61-butyric acid methyl ester (PCBM, 99.9%) were purchased from Advanced Election Technology Co., Ltd. Fullerene (C_60_, 99%) is purchased from Vizuchem Co., Ltd. Florine-doped tin oxide (FTO-coated glass, square resistance 15 Ω) is purchased from Advanced Election Technology Co., Ltd. 4-(7H-dibenzo[c,g]carbazol-7-yl)butyl)phosphonic acid (4PADCB, 99%) is purchased from TCI. Other materials were purchased from Alfa Aesar. All materials were used as received and stored inside a nitrogen glovebox.

### Preparation of Perovskite Precursor

The 1.3 M Cs_0.22_FA_0.78_Pb(I_0.85_Br_0.15_)_3_ WBG perovskite precursor was prepared by introducing stoichiometric ratios of PbI₂, PbBr₂, CsI, and FAI and 2 wt% PbCl₂ and 10 wt% MACl dissolved in 1 mL of mixed solvents. The solvent systems were designed as either: (1) DMF:DMSO (4:1 v/v), or (2) GVL + DMF:DMSO with fixed 4:1 v/v ratio, where DMF/GVL volume ratios were varied (9:1, 8:2, 7:3). The mixture was stirred overnight at room temperature within a nitrogen glovebox. Prior to use, the precursor solution was filtered through a 0.45 μm polytetrafluoroethylene syringe filter.

### Fabrication of Single-Junction Wide Bandgap Perovskite Devices

The FTO glass substrates were sequentially cleaned with acetone, isopropanol, ethyl alcohol, and deionized water in a sonication bath for 30 min, respectively. Then, the substrates were dried by flowing nitrogen gas, followed by treatment in an ultraviolet-ozone chamber for 15 min. A 26-nm-thick NiOx layer was deposited via electron beam evaporation. Subsequently, a 0.5 mg mL^−1^ solution of 4PADCB was spin-coated onto the substrate at 3000 rpm for 30 s, followed by annealing at 100 °C for 10 min. Then, 100 uL perovskite solution was uniformly dispensed onto the FTO/NiOx/4PADCB substrate. The spin-coating process was conducted in two stages:1000 rpm for 10 s, 4000 rpm for 25 s. During the final 10 s of the spin-coating process, 150 μL of BAC was dripped onto the rotating substrate. Then, the perovskite films were immediately placed on a hot plate and annealed at 120 °C for 30 min. Then, PCBM (20 mg mL^−1^ in chlorobenzene) and BCP (saturated isopropanol solution) were spin-coated with spinning speeds of 4000 rpm for 30 s and 5000 rpm for 30 s, respectively. Lastly, 100 nm Ag was evaporated under high vacuum (< 8 × 10^–4^ Pa) on the substrates to form electrodes.

### Fabrication of Tandem Perovskite/Silicon Devices

Silicon heterojunction (SHJ) bottom cells were fabricated on double-side textured n-doped wafers. The thicknesses of ITO recombination junction were set as 20 nm by sputtering. The 40-nm NiO_X_ is deposited by electron beam evaporation as HTL. And, the spin-coating method for 4PADCB remained identical to the aforementioned procedure, with the concentration increased to 1 mg mL^−1^. The deposition procedures of the perovskite layer were in consistence with the single-junction device deposition recipe with a 1.8 M of the solution. 30 nm of C_60_ was thermally evaporated as ETL on top of the perovskite layer. A buffer layer of SnO_2_ (250 cycles) was then deposited by the thermal atomic layer deposition (ALD) technique. Subsequently, 100-nm indium zinc oxide (IZO) top electrode with a sheet resistance of 80 Ω per square was deposited via sputtering at room temperature. Silver with thickness of 300 nm was thermal evaporated around the IZO and the p-side ITO through a shadow mask, respectively. The aperture area was calculated to 1 cm^2^, deducting the area of the two thin grids. At last, 100 nm of MgF_2_ antireflection films were added through thermally evaporated at a rate of 2.5 Å s^−1^ to further enhance the light absorption.

### Device Characterization

The dynamic light scattering of the perovskite precursor solutions was analyzed using a Zetasizer Nano laser particle size analyzer. X-ray diffraction (XRD) measurements were obtained by using a SmartLab. Grazing-incidence wide-angle X-ray scattering (GIWAXS) was performed by Xeuss.2.0. The morphology of the films was characterized by field emission SEM (SU8020). UV–vis absorption spectra were recorded using a U-4150 Hitachi UV–vis spectrophotometer through transmittance. Photoluminescence spectra (PL) and time-resolved photoluminescence (TRPL) were measured on a fluorescence spectrometer (Pico1000, Light-Stone CO, LTD) using the time-correlated single photon counting technique. The conductive atomic force microscopy (c-AFM) was performed on an instrument of Bruker Nanowizard 4XP. The obtained data were processed and analyzed using NanoScope Analysis 1.9 software. The water contact angles of the perovskite films were characterized using a DSA100 contact angle goniometer. SCLC measurements were obtained using a Keithley 2450 Source Meter under dark conditions, and devices were tested in forward scan direction 0–3 V with step size of 0.005 V. The transient photovoltage (TPV) measurements were measured using a potentiostat (ENIMIUN, Zahner, Germany). Mott–Schottky and electrochemical impedance spectroscopy (EIS) measurements were performed using an impedance spectroscopy analyzer (ZENNIUM, Zahner, Germany). The *J*–*V* measurement was performed using a Keithley 2450 Source Meter under AM 1.5G illumination from an Oriel 9600 solar simulator under air condition. A standard crystalline silicon cell was used to calibrate the light intensity of solar simulator before measurement. For WBG PSC, the devices were measured with a scan rate of 0.02 V s^−1^ and a delay time of 1 ms with the reverse scan range from 1.3 to − 0.1 V and the forward scan from − 0.1 to 1.3 V. Meanwhile, tandem perovskite/silicon devices were tested in the reverse scan direction of 2.05 to − 0.2 V and forward scan direction of − 0.2 to − 2.05 V in 0.02 V steps. The external quantum efficiency (EQE) spectra were obtained by using a QE system (EnliTech).

### DFT Calculations

#### Calculating Binding Energy

The research utilized the CASTEP module within the Materials Studio software to conduct first-principle calculations [39]. The computations relied on density functional theory (DFT). The electronic-ion interactions were approximated with the On-the-fly generated (OTFG) ultrasoft pseudopotential, and the exchange–correlation term was addressed using the generalized gradient approximation (GGA) within the Perdew–Burke–Ernzerhof (PBE) functional [40]. A plane wave energy cutoff of 500 eV was applied. The crystal structure underwent geometric optimization using the Broyden–Fletcher–Goldfarb–Shanno (BFGS) algorithm, with convergence criteria set at 2 × 10^–5^ eV atom^−1^, 0.01 eV Å^−1^, 0.1 GPa, and 0.002 Å for atomic forces, single atom energy, lattice stress, and maximum atomic displacement. These parameters ensured the geometric convergence of the crystal structure. Structural optimization employed the GGA-PBE functional to eliminate strain energy and relax the structure. The binding energy was calculated using the following formula:

$${E}_{b} = {E}_{\mathrm{t}\mathrm{o}\mathrm{t}\mathrm{a}\mathrm{l}} - {E}_{\mathrm{c}\mathrm{a}\mathrm{t}\mathrm{i}\mathrm{o}\mathrm{n}} - {E}_{\mathrm{s}\mathrm{o}\mathrm{l}\mathrm{v}\mathrm{e}\mathrm{n}\mathrm{t}}$$

where ${E}_{\mathrm{t}\mathrm{o}\mathrm{t}\mathrm{a}\mathrm{l}}$, ${E}_{\mathrm{c}\mathrm{a}\mathrm{t}\mathrm{i}\mathrm{o}\mathrm{n}}$, and ${E}_{\mathrm{s}\mathrm{o}\mathrm{l}\mathrm{v}\mathrm{e}\mathrm{n}\mathrm{t}}$ represent the total energy of the solvent combined with the corresponding perovskite precursor cation, the energy of the cation, and the energy of the solvent, respectively.

#### Calculating Adsorption Energy

To investigate the interaction between γ-valerolactone (GVL) and various crystal surfaces of formamidinium lead iodide (FAPbI_3_), we performed binding energy calculations on the (100), (110), and (111) planes using density functional theory (DFT) as implemented in Materials Studio CASTEP (version 2017). Each surface was modeled using a periodic slab with a thickness of approximately 18.67 Å, sufficient to represent the surface while minimizing interactions between periodic images. The calculations employed the Perdew–Burke–Ernzerhof (PBE) exchange–correlation functional within the generalized gradient approximation (GGA), along with on-the-fly generated (OTFG) ultrasoft pseudopotentials. A plane wave energy cutoff of 490 eV was used. The self-consistent field (SCF) tolerance was set to 2 × 10^–6^ eV, with convergence criteria for geometry optimization defined as 2 × 10^–5^ eV atom^−1^ for energy, 0.05 eV Å^−1^ for maximum force, and 0.1 GPa for maximum stress. Spin polarization was not enabled, although the system included an odd number of electrons, which triggered warnings suggesting possible spin asymmetry. Binding energies were calculated by comparing the total energy of the GVL-adsorbed slab to the sum of the energies of the isolated surface and the GVL molecule.

## Results and Discussion

### Machine Learning Analysis

**Fig. 1:**
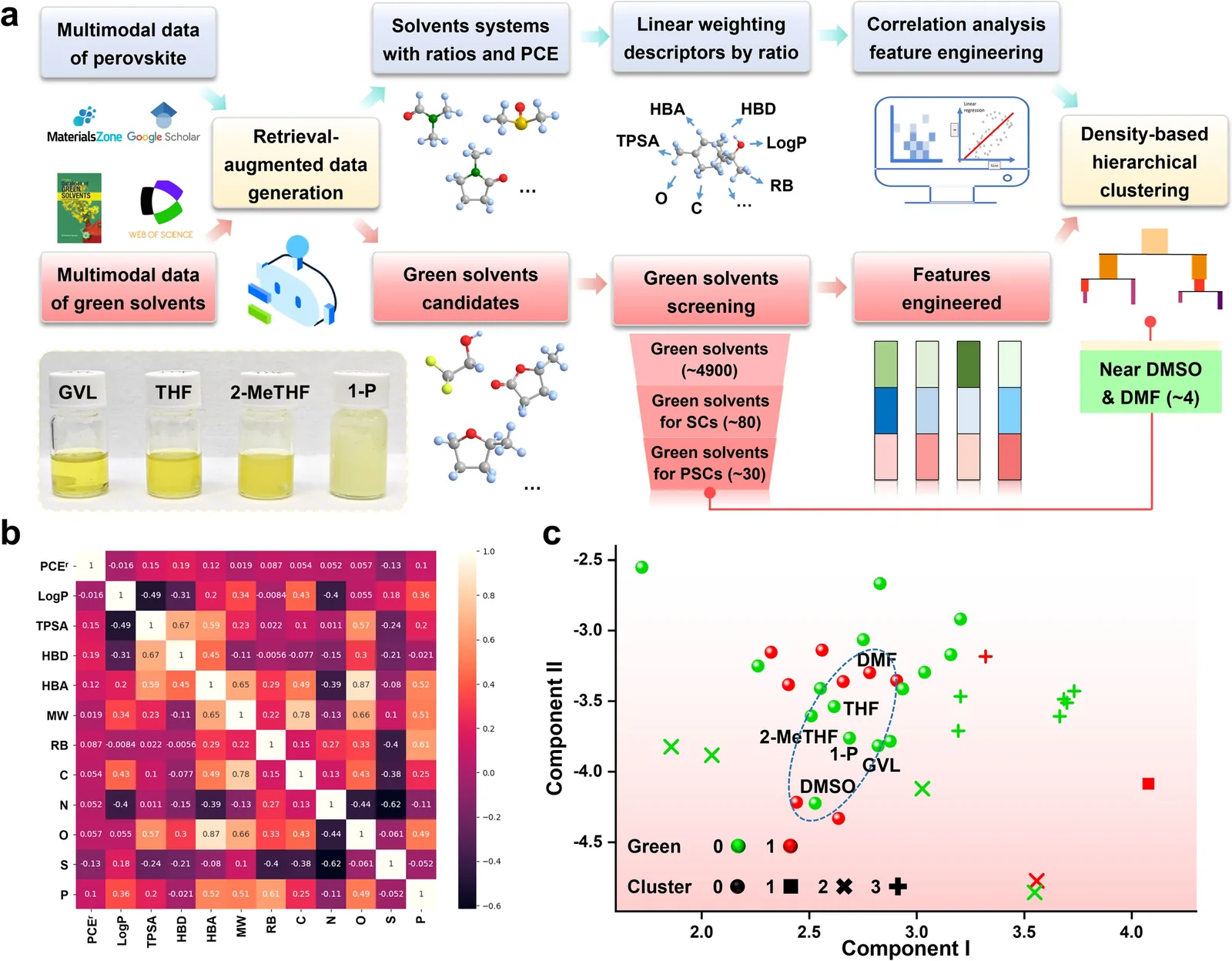
**a** Workflow of screening the green solvent candidate from multimodal data. **b** Correlation matrix between PCE residual (PCE^r^, the PCE value that has been stripped of the influence of area using the linear regression method) and other features. **c** Distribution and clustering visualization of the perovskite solvents using t-SNE and HDBSCAN. Components I and II mean dimension-1 and dimension-2 after dimensionality reduction using t-SNE, respectively. Insert picture presents the solubility of 1.8 M perovskite precursor with the co-solvent of GVL, THF, 2-MeTHF and 1-P

Figure 1a shows the workflow of screening the functionalized and green solvent candidate from multimodal data. To identify the novel solvent substitute, we gathered multimodal data on PSCs from material databases (Materials Zone) and literature sources (Web of Science and Google Scholar), including text, tables, and figures. To extract precise and detailed information about co-solvent systems and their associated PCE, we utilized the retrieval-augmented generation (RAG) technique to process this extensive dataset. This approach is promising as it can retrieve up-to-date information from external document repositories, breaking through the knowledge limitations in the fixed parameters of the language model. In contrast to the pure language model, RAG also reduces fictions or errors in the generated content by retrieving real documents [41]. Therefore, it is especially suitable for answering factual questions, such as technical documents, legal texts, and academic materials. The frozen LLM we employed in the RAG LLM agent is the generative pre-trained transformer 4 (GPT-4) model [41]. RAG adopts a retrieve-read framework, which is not only straightforward but also highly efficient for this task. With this technique, we identified 270 unique co-solvent systems in PSCs from more than 8000 solvents. To obtain a comprehensive characterization of solvent molecules, we selected as many relevant features as possible that are accessible through RDKit (version 2024.03.6) and significant for solvent properties. RDKit is a powerful open-source cheminformatics toolkit that offers a range of effective functionalities for the calculation of molecular descriptors. As illustrated in Fig. 1b, we systematically compiled 13 chemical descriptors, referred to as features, for each solvent. To accurately represent a co-solvent system, these features were initially weighed linearly based on ratios corresponding to each solvent component, which follows the Law of Mixtures. The definitions of the abbreviations are provided in Table S1. Specifically, hydrogen-bond donor (HBD) and hydrogen-bond acceptor (HBA) values represent the potential electron-donating ability and the number of coordination sites, whereas the oil–water partition coefficient (logP) and topological polar surface area (TPSA) reflect polarity and ionic stability. Molecular weight can partially indicate the solvent’s boiling point. These descriptors are widely considered to be closely associated with the crystallization process of perovskite materials [42]. HBD and HBA can modulate the interaction strength between the solvent and perovskite nanocolloids, thereby influencing the nucleation rate and growth rate of perovskite [43]. The logP and TPSA of solvent affect the interfacial energy of the perovskite solution, which in turn impacts the nucleation energy barrier and nucleation rate. These four parameters—HBD, HBA, logP, and TPSA—also influence the crystal facet energy of perovskite, ultimately optimizing the exposed crystal facets in the final perovskite thin film. It should be noted that boiling point and donor number (D_N_) values were excluded because the boiling points reported in databases are not consistently measured under standard pressure, and there is currently no accessible database providing reliable D_N_ values. Subsequently, a correlation analysis was conducted between these features and the PCE. Through feature engineering, we selected the features that exceeded a predefined correlation threshold. For each solvent, we derived its engineered feature vectors. We hypothesized that any solvent with engineered features closely resembling those of DMSO and DMF could serve as a potential candidate for replacing or partially substituting DMF and regulating crystallization dynamics. Given that the engineered features are correlated with PCE, solvents with similar features to DMSO and DMF are likely to achieve high PCE.

To also identify green solvents, we systematically collected multimodal data on green solvents from green solvent handbooks and the Web of Science database. By leveraging the RAG approach once again, we constructed a comprehensive dataset comprising approximately 4900 green solvents, along with their detailed properties and application descriptions. Subsequently, we employed keyword-based screening to identify around 30 green solvents that exhibit strong correlations with PSCs. To further screen these solvents, we assigned engineered features and applied density-based hierarchical clustering using the hierarchical density-based spatial clustering of applications with noise (HDBSCAN, minimum cluster size = 5) algorithm to categorize them into distinct groups. This method demonstrates superior performance compared to centroid-based clustering techniques (e.g., K-means), particularly for high-dimensional, non-Gaussian distributed datasets [44]. To visualize the clustering results, we applied t-distributed stochastic neighbor embedding (t-SNE) for dimensionality reduction. As described in Fig. 1c, we took DMF and DMSO as the foci of the ellipse. The solvents located within this ellipse are regarded as neighbors of both DMSO and DMF, and are therefore selected as potential candidates, including GVL, tetrahydrofuran (THF), 2-methyltetrahydrofuran (2-MeTHF), and 1-pentanol (1-P).

As mentioned, the fabrication of monolithic perovskite/silicon TSCs requires a high-concentration perovskite precursor solution to thicken the perovskite layer capping the silicon pyramids. Thus, we evaluated the dissolving capability of four solvent candidates by preparing a 1.67-eV perovskite precursor solution with the concentration as high as 1.8 M. As shown in the inset of Fig. 1, after stirring overnight, only the co-solvent system composed of GVL/DMF/DMSO could completely dissolve the precursors. THF and 2-MeTHF failed to dissolve the perovskites due to their low D_N_ values (Table S2). Additionally, the active proton in 1-P enables the formation of hydrogen bonds with DMF/DMSO, weakening the interaction between the perovskites and the host solvent system. Therefore, we selected GVL as the co-solvent for the 1.67-eV WBG perovskites and proceeded with further studies.

### Effect of GVL on Precursor Solution and Perovskite Films

**Fig. 2:**
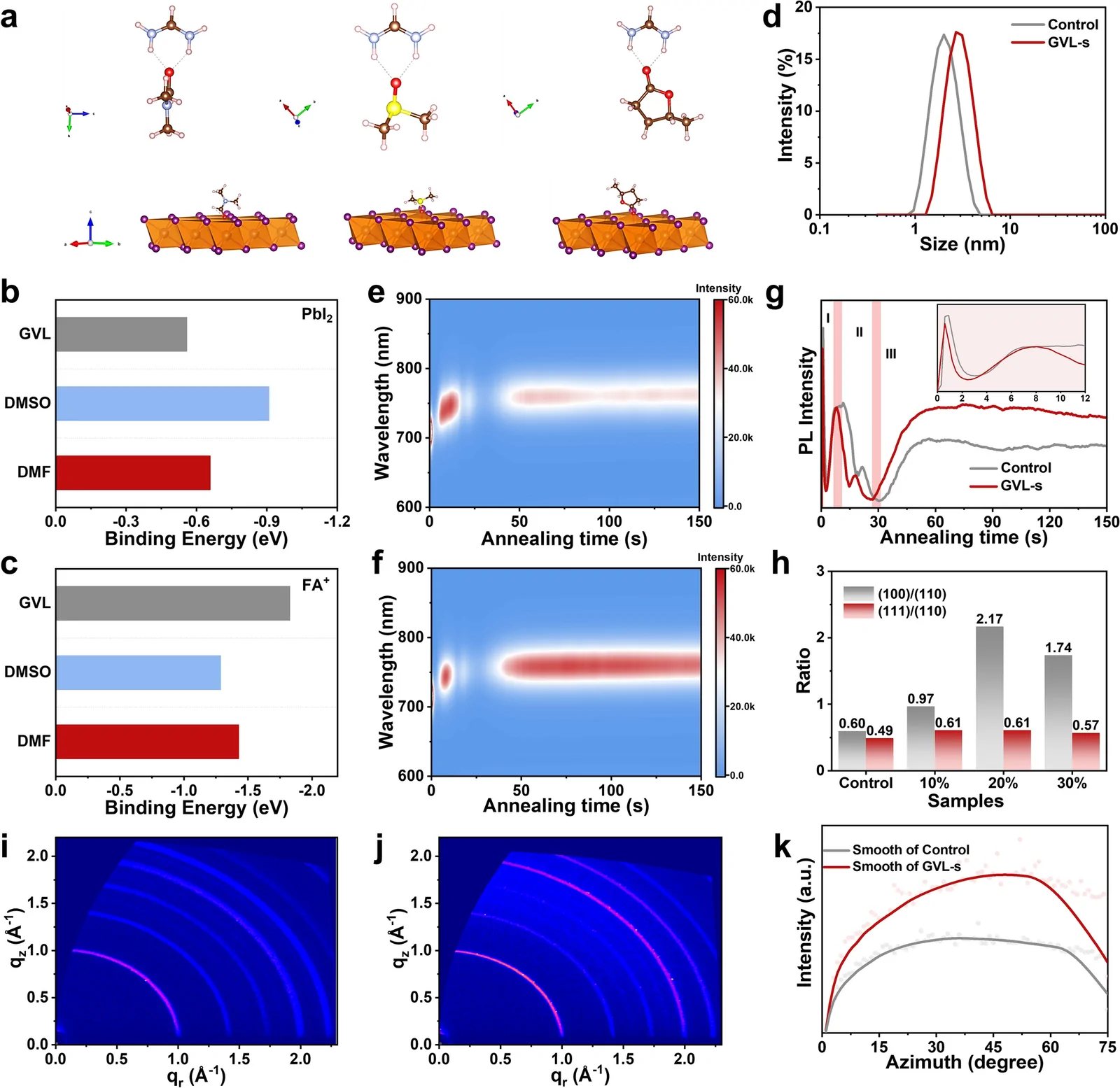
**a** Molecular structures of FA^+^·DMF, FA^+^·DMSO, FA^+^·GVL,·PbI_2_·DMF, PbI_2_·DMSO, and PbI_2_·GVL. Average binding energies of **b** PbI_2_ and **c** FA^+^ with solvents. **d** Colloid size distributions of control and GVL-based precursor solutions after filtration. In situ PL peak intensity of **e** control- and **f** GVL-based perovskite films during crystallization. **g** Time evolution of PL intensity for control- and GVL-based films. **h** Ratio of the (100) and (111) planes to the (110) plane at different samples. GIWAXS patterns of **i** control- and **j** GVL-based perovskite films. **k** Intensity azimuthal pole figures of control- and GVL-based perovskite films

Density functional theory (DFT) calculations were conducted to systematically to analyze the interaction between GVL and perovskite precursors, while DMF and DMSO were employed as references. The optimized configurations, as shown in Fig. 2a, reveal hydrogen bonding between FA⁺ and the respective solvents. As illustrated in Fig. 2b, GVL exhibits a significantly stronger interaction with formamidine (FA⁺), with a binding energy of − 1.83 eV, compared to − 1.43 eV for DMF and − 1.29 eV for DMSO. This result aligns with the hard-soft acid–base principle, as FA^+^ acts as a soft Lewis acid, whereas GVL serves as a softer Lewis base compared to DMF and DMSO [45]. The binding energies between PbI_2_ and the oxygen atoms of DMF, DMSO, and GVL were calculated to be –0.66, –0.91, and –0.56 eV, respectively (Fig. 2c). These results indicate that PbI_2_ exhibits the strongest interaction with DMSO and the weakest with GVL, in line with lower D_N_ of GVL and its weaker coordination ability with Pb^2+^ [46]. Subsequently, we systematically investigated the influence of GVL on the microstructure of perovskite precursor solutions, the crystallization kinetics process, and the final crystal quality and anisotropy, aiming at comprehensively revealing the influencing mechanism of GVL on the crystallization behavior [47]. In the experiment, the volume ratio of GVL + DMF to DMSO was fixed at 4:1. A mixture of DMF/GVL with a composition of 8:2 (denoted as 20 vol%) was utilized in the study. For simplicity, the conventional solution system of DMF/DMSO was designated as the control group and the group that GVL substituting partial DMF was abbreviated as “GVL-s”. Figure 2d shows the dynamic light scattering (DLS) spectra of the precursor solutions with and without GVL. The colloid size increases to 2.67 nm upon the addition of GVL, compared to approximately 1.94 nm in the DMF/DMSO solution system. This colloidal aggregation can be attributed to the reduced solvation capability of GVL with a low D_N_. In large colloids, the internal ionic arrangement becomes more ordered, approaching the short-range structural characteristics of the final crystalline phase [48]. This increased structural order suggests enhanced quasi-solid behavior and is likely accompanied by an increased effective solid–liquid interfacial energy (*γ*_SL_) [49, 50]. According to Eq. (1) in classical nucleation theory,

1 $$\Delta {G}^{*}=\frac{16\pi {{\gamma }^{3}}_{SL}}{3{\left|\Delta {G}_{\vartheta }\right|}^{2}}$$

where Δ*G*_*v*_ represents the change in Gibbs free energy per unit volume during the phase transition, it can be deduced that the nucleation Gibbs free energy barrier, Δ*G*^∗^, will increase [51]. This, in turn, leads to a reduction in nucleation density, thereby facilitating an increase in the crystal grain size of the final perovskite film [52].

Figure 2e, f shows the in situ photoluminescence (PL) spectra of the crystallization dynamic process of perovskite films with a time resolution of 300 ms. We placed the spin-coated wet films on a hot plate of 80 °C and recorded the changes in PL spectra over time. Figure 2g extracts the change in PL intensity as a function of time. The evolution of PL intensity can be divided into three distinct stages. In Stage I, the initial decrease in PL intensity corresponds to the partial dissolution of the initial crystal nuclei formed in the wet films. This is due to the surface nucleation mechanism of perovskite films, where the evaporation of residual solvents during the heating process dissolves the surface nuclei. The subsequent recovery of PL intensity in Stage I indicates the formation of new nuclei, and their density gradually increases. The enlarged image (the inset) of Stage I shows that the initial PL intensity of the wet film containing GVL is lower than that of the control sample, indicating a lower nucleation density, which is consistent with DLS results. In Stage II, the decrease in PL intensity indicates that the solvent is gradually exhausted, exposing the grain boundaries between the nuclei, which leads to non-radiative recombination. During this stage, the PL intensity of the GVL-containing film decreases more quickly, suggesting a higher solvent evaporation rate, which is attributed to the weaker interaction between GVL and lead halide. This, in turn, also facilitated the Ostwald ripening process in Stage III, as indicated by the earlier onset of increased PL intensity. The final perovskite thin film containing GVL ultimately shows higher PL intensity, indicating a lower defect density in the film. Therefore, GVL can modulate crystallization dynamics by suppressing the nucleation process, accelerating solvent deabsorption kinetics, and enhancing Ostwald ripening, ultimately yielding perovskite films with reduced defect density [53].

The morphology and crystallinity of perovskite films prepared with varying GVL ratios were systematically investigated to clarify its impact on the final film properties. As shown in Fig. S1**a**, the control films exhibit an average grain size of 192 nm with a high density of grain boundaries (GBs), indicative of numerous structural defects that hinder charge transport and promote carrier recombination [54]. The incorporation of GVL leads to increased average grain sizes, from ~ 200 nm at 10 vol% to ~ 247 nm at 20 vol% (Fig. S1b, c), representing reduced defect density. However, at 30 vol% GVL, the grain size decreases to 154 nm and the film surface appears blurred, suggesting deterioration in film quality (Fig. S1d). Figure S2a–c presents photographs and scanning electron microscopy (SEM) images of the buried surfaces of perovskite films obtained by delaminating the perovskite layers from their substrates. The control film exhibits numerous voids and a non-uniform morphology, whereas the GVL-based film shows a dense, void-free surface. This improvement is attributed to the weaker binding between GVL and lead halide, which suppresses the formation of the solvate phase at the buried interface during crystallization. Such a morphology is expected to facilitate more efficient hole injection and enhance interfacial stability. These morphological evolutions underscore the effectiveness of GVL as a solvent for controlling perovskite crystallization and optimizing film quality.

The X-ray diffraction (XRD) patterns of the perovskite films shown in Fig. S3 reveal that the intensity of the diffraction peaks initially increases and then decreases as the volume ratio of GVL rises. The peak intensity reaches its maximum at 20 vol% GVL, which is consistent with the SEM results. Interestingly, in the control sample, the (110) plane is preferentially oriented, while the preferential crystal plane gradually shifts to the (100) plane as the GVL ratio increases. Figure 2h quantifies the ratios of the (100) and (111) planes to the (110) plane at various GVL ratios, showing that in the sample with 20 vol% GVL, the (100) and (111) planes become the most preferential. Previous studies have shown that carrier mobility is similar for the (100) and (111) planes, both of which are significantly higher than for the (110) plane [55]. Additionally, the (111) plane contributes superior stability to perovskites [56]. This suggests that the preferential crystal plane induced by GVL will enhance both the electrical performance and stability of the films. To investigate the mechanism behind this alteration in preferential planes, we conducted density functional theory (DFT) calculations to estimate the adsorption energies (E_ads_) of GVL molecules on various crystal facets of perovskites. As shown in Fig. S4, the calculated adsorption energies of GVL on the (100), (110), and (111) facets are − 2.19, − 2.55, and − 1.92 eV, respectively, indicating stronger binding on the (100) and (110) facets compared to the (111) facet. This result supports the preferential growth of perovskites along the (100) and (110) surfaces [57]. Furthermore, we compared the grazing incidence wide-angle X-ray scattering (GIWAXS) patterns of the control and 20% GVL perovskite films. As per Fig. 2i, j, the GVL-containing films exhibit more intense diffraction rings, suggesting enhanced crystallinity. To quantitatively analyze the orientation distribution, we integrated the intensity along the Debye–Scherrer ring of the (200) plane (*q* ≈ 2 Å⁻^1^) and plotted its dependence on the azimuthal angle. As shown in Fig. 2k**,** the GVL-based films show a more pronounced preferential orientation compared to the control. This characteristic will improve carrier transport and reduce the density of trap states, thereby enhancing the overall property of the perovskite films [58]. Collectively, these results demonstrate that GVL effectively modulates the crystallization behavior of perovskite films, leading to enhanced morphology, crystallinity, and crystal orientation.

### Single-Junction WBG PSCs and Perovskite/Silicon TSCs

**Fig. 3:**
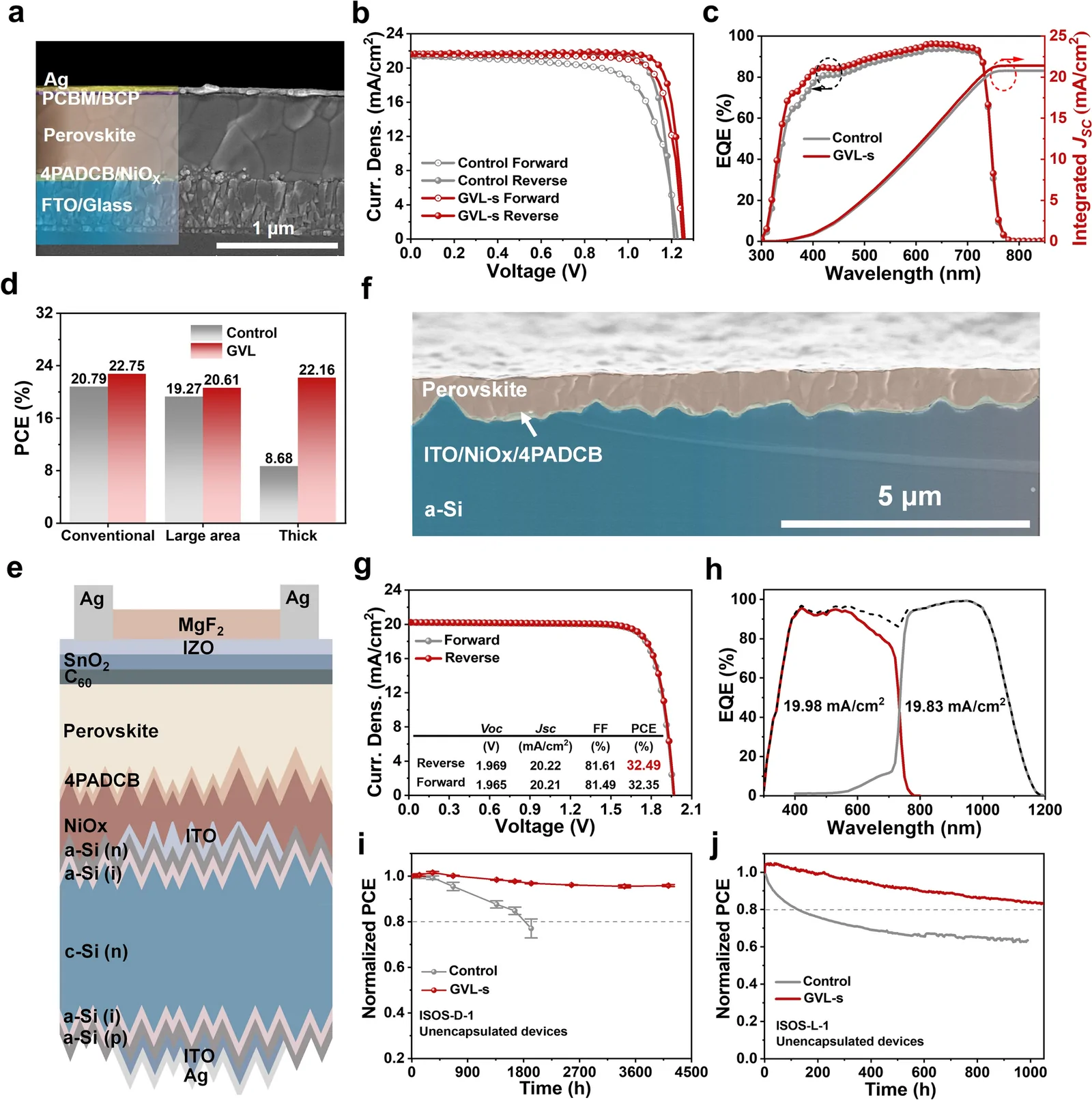
**a** Cross-sectional SEM image of single-junction PSC. **b** *J − V* curves of champion devices measured under forward and reverse scanning. **c** EQE spectra and the corresponding integrated *J*_SC_ of control and GVL PSCs. **d** Histogram of the average PCE with and without GVL-based conventional, large-area, and thick films PSCs. **e** Schematic diagram of perovskite/silicon TSC. **f** Cross-sectional image of perovskite layers on textured silicon substrates. **g** *J*–*V* curves of the champion tandem device. **h** EQE spectra of a current-matched monolithic perovskite/silicon TSC. **i** Shelf stability of unencapsulated devices;** j** Long-term continuous MPP tracking under 1-sun illumination of the control and GVL devices

To evaluate the impact of GVL on the photovoltaic performance of devices, we fabricated planar heterojunction WBG PSCs with an FTO/NiOx/4PADCB/perovskite/PCBM/BCP/Ag architecture. In this structure, 4PADCB refers to [4-(7H-dibenzo[c,g]carbazol-7-yl)butyl]phosphonic acid, PCBM is [6, 6]-phenyl-C61-butyric acid methyl ester, and BCP denotes bathocuproine. Figure 3a shows the cross-sectional SEM image of PSC with labeled functional layers. As illustrated in Fig. S5a-d, the photovoltaic performance of the control and GVL-based devices was statistically evaluated based on 15 individual devices. Compared to the control, the incorporation of GVL significantly improves the photovoltaic parameters, particularly the open-circuit voltage (*V*_OC_) and fill factor (FF). The devices achieve peak performance at the GVL volume ratio of 20 vol%, consistent with previous observations. The control devices yield an average PCE of 20.79% ± 0.59%, whereas the GVL-based devices demonstrate a notably higher average PCE of 22.75% ± 0.32%. Moreover, the narrower distribution is also indicative of improved reproducibility and reliability. Figure 3b shows the *J*–*V* curves of the champion control and (20% vol) GVL-based devices. Detailed photovoltaic performance parameters are listed in Table S3. The control device achieves a champion PCE of 21.79%, with a *V*_OC_ of 1.210 V, *J*_SC_ of 21.34 mA cm^−2^, and FF of 84.34%. In comparison, the GVL-based device demonstrates significantly reduced hysteresis and achieves a champion PCE of 23.30%, with an impressively high *V*_OC_ of 1.257 V, *J*_SC_ of 21.64 mA cm^−2^, and FF of 85.63%. We made an explicit comparison with recently reported cosolvent systems applied in WBG PSCs (Table S4). Both the PCE and *V*_OC_ × FF in this work rank among the highest values reported for 1.67-eV PSCs (Table S5 and Fig. S6a, b for the literature summary). The steady-state output efficiencies were measured at the maximum power point (MPP), as depicted in Fig. S7. The GVL-based device achieves a stable output efficiency of 22.9% (*V*_MPP_ = 1.12 V), significantly surpassing the 19.2% (*V*_MPP_ = 1.06 V) of the control devices. The integrated *J*_SC_ from the external quantum efficiency (EQE) curves of the control and GVL-based devices are 20.80 and 21.41 mA cm^−2^, respectively, which is consistent well with *J–V* measurements (Fig. 3c). The GVL-based device demonstrates superior external quantum efficiency (EQE) across the entire spectral range, particularly within the 350 to 450 nm region. This enhancement is consistent with the improved quality of the buried interface and the increased crystallinity of the films.

In the fabrication of laboratory-scale perovskite/crystalline silicon TSCs, perovskite films with an area of 1 cm^2^ and a thickness of approximately 1 μm are required to uniformly cover the pyramids on silicon substrates [59]. However, achieving precise control over film uniformity, thickness, and crystallinity during the solution-based fabrication process remains a considerable challenge. This is primarily because the defect density tends to increase significantly with the enlargement of the active area and the thickening of the film. To validate the effects of GVL on the uniformity of perovskite films, we fabricated planar PSCs with an active area of 1 cm^2^. The control device yields a PCE of 19.60%, while the GVL device delivered a champion PCE of 21.11%, with a *V*_OC_ of 1.215 V, a *J*_SC_ of 21.63 mA cm⁻^2^, and an FF of 80.37% (Fig. S8). It appears to be one of the highest PCE reported for 1-cm^2^ 1.67 eV-PSCs (Table S6 and Fig. S9 for the literature summary). As per Fig. S10, the GVL-based PSCs achieved a steady-state output efficiency of 20.3%, notably higher than the 18.4% recorded for the control devices. To evaluate device reproducibility, 11 individual 1-cm^2^ PSCs were fabricated for each group (Fig. S11). The GVL-based devices exhibited a narrower PCE distribution with an average of 20.61% ± 0.39%, clearly demonstrating that the incorporation of GVL significantly improves the uniformity of perovskite films. Subsequently, we investigated the influence of GVL on the thickness tolerance of perovskite films. The concentration of the precursor solution was increased to 1.8 M to achieve a film thickness of ~ 1 μm and the cross-sectional SEM of films is depicted in Fig. S12. The corresponding *J*–*V* curves of champion PSCs are shown in Fig. S13. The champion GVL-PSC deliver a *V*_OC_ of 1.241 V, a *J*_SC_ of 21.59 mA cm^–2^ and an FF of 84.42%, resulting in a PCE of 22.61%. By contrast, the control device showed a much lower PCE of 13.18%, with a lower *V*_OC_ of 1.147 V and FF of 57.21%, due to the poor film quality. The statistic photovoltaic parameters of GVL-based devices with 18 individuals for are presented in Fig. S14. The average PCE reaches 22.16% ± 0.35% with a narrow distribution, highlighting the excellent reproducibility of GVL-based devices in comparison with the control samples. Figure 3d compares the PCE distribution of PSCs based on three types of perovskite films: conventional (0.09 cm^2^ area and 673 nm thickness), large area (1 cm^2^), and thick (approximately 1 μm), prepared with or without GVL. The results highlight that GVL significantly improves film uniformity and thickness tolerance attributed to the optimization of crystallization dynamics, which are both essential for achieving high-efficiency perovskite/silicon TSCs [7].

In further, the GVL solvent was employed to prepare monolithic perovskite/silicon TSCs on the basis of double-sided nanotextured crystalline silicon solar cells (Fig. S15). The schematic diagram of the complete tandem architecture is presented in Fig. 3e. As shown in Fig. 3f, the perovskite film prepared with 1.8 M solution fully covers the pyramids, and the perovskite film exhibits large grains that extend vertically across the silicon substrate. This comprehensive coverage and improved crystallinity will contribute to better charge transport and reduced recombination rate. Figure 3g presents the *J*–*V* curves of the champion TSCs (1 cm^2^), which exhibits a PCE of 32.49%, with a *V*_OC_ of 1.969 V, a *J*_SC_ of 20.22 mA cm^−2^, an FF of 81.61%, and negligible hysteresis. Figure 3h displays the EQE spectra of the tandem device, where the *J*_SC_ values for the perovskite and silicon sub-cells are 19.98 and 19.83 mA cm⁻^2^, respectively. This not only demonstrates the current match between sub-cells but is also consistent with the *J*–*V* measurements. The champion device also maintains a stabilized PCE of 32.3% over 300 s (Fig. S16), indicating excellent operational stability. Moreover, Fig. S17 illustrates the PCE distribution of 16 devices, confirming that the application of GVL in the perovskite precursor solution ensures high device reproducibility.

In addition to device performance, the effect of GVL on the long-term stability of single-junction was further examined. For the shelf-life stability measurement, the unencapsulated PSCs were stored at room temperature with 20–30% relative humidity, in accordance with the ISOS-D-1 protocol. Figure 3i shows that both control and GVL-based PSCs exhibited an initial rise in PCE, likely resulting from enhanced interfacial contact and air-induced passivation [60], followed by a gradual degradation. The GVL-based devices exhibit a T_80_ lifetime (time to retain 80% of the initial PCE) exceeding 4000 h, significantly outperforming the control devices, which show a T_80_ of ~ 1900 h. The primary cause of PCE degradation is the notable decline in FF (Fig. S18), indicating that the perovskite layer processed with GVL possesses superior resistance to moisture-induced deterioration [61]. This enhanced stability is further corroborated by the larger Wenzel model contact angle of 56.9° observed on the GVL-perovskite surface, compared to 40.7° for the control (Fig. S19) [62, 63]. Figure S20 shows the XRD patterns of perovskite films stored under ambient conditions for 13 days to analyze the degradation mechanism. The control film exhibits a decrease in peak intensity along with the appearance of *δ*-CsPbI_3_, *δ*-FAPbI_3,_ and PbI_2_ diffraction peaks, indicating partial phase transformation and decomposition caused by moisture penetration through grain boundaries and interfaces. In contrast, the GVL-based film shows no signs of phase transformation or PbI_2_ peaks, which can be attributed to the improved film quality and reduced grain boundary density. Figure 3j presents the MPP tracking stability of unencapsulated devices under continuous one-sun illumination, following the ISOS-L-1 protocol. The GVL-based PSC exhibits excellent operational stability, achieving a T_80_ lifetime exceeding 1000h under continuous one-sun illumination (ISOS-L-1), significantly surpassing the control device, which exhibits a T_80_ lifetime of only 375 h. This enhanced photostability is attributed to the reduced charge defect density and suppressed vacancy-assisted ion migration in the GVL-based perovskite films.

### Mechanism of GVL Improving the Performance of Devices

**Fig. 4:**
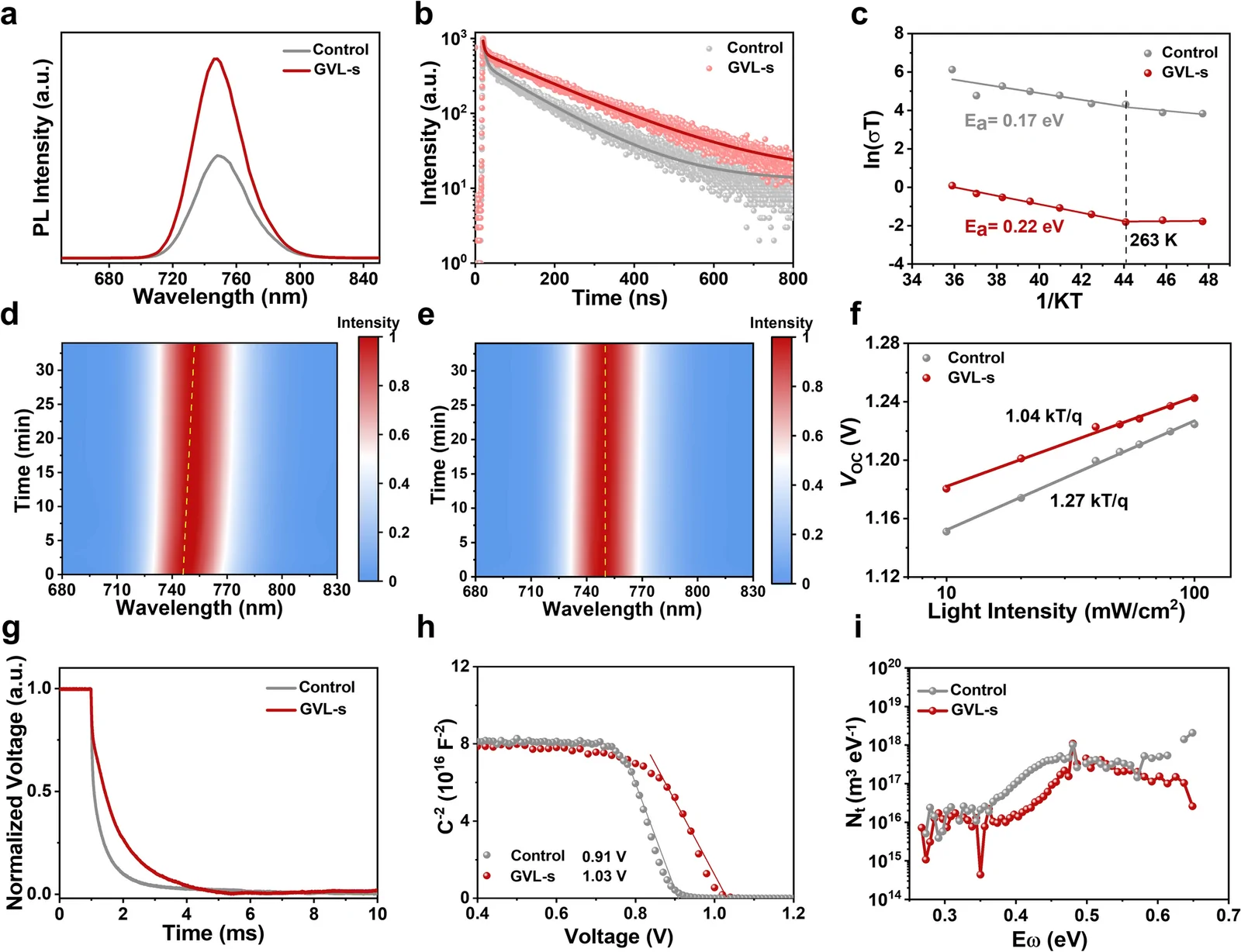
**a** PL and **b** TRPL measurements of the control and GVL-based perovskite films. **c** Temperature-dependent conductivity of control and GVL-based perovskite films. The devices with the structure of FTO/perovskite/Au were used. PL peak evolution over time (under continuous illumination with a 520 nm) laser of **d** control and **e** GVL-based perovskite films. **f** *V*_OC_ *versus* light intensity;** g** TPV curves; **h** Mott–Schottky curves; **i** *N*_t_ obtained from TAS measurement of control and GVL-based PSCs

Steady-state photoluminescence (PL) and time-resolved photoluminescence (TRPL) measurements were conducted to assess the impact of GVL on carrier recombination dynamics. Figure 4a shows that the PL intensity of the GVL-optimized perovskite film is significantly higher than that of the control, indicating that GVL effectively suppresses non-radiative recombination and contributes to an increase in the quasi-Fermi level splitting. The carrier lifetimes were obtained by fitting the TRPL decay curves in Fig. 4b using a biexponential function, and the corresponding values are summarized in Table S7. The prolonged carrier lifetime in the GVL-based film is attributed to the reduction in the density of grain boundaries. In addition, the higher *τ*_2_ value indicates fewer bulk Schottky defects, which is consistent with the improved crystallinity in the GVL-perovskite films [64]. For WBG perovskites, reducing defect density not only optimizes the carrier recombination dynamics but also suppresses ion migration, thereby alleviating the photo-induced phase segregation process [65–67]. To verify this deduction, as per Fig. 4c, we studied ion migration behavior by measuring the temperature-dependent conductivity of perovskite films. The result reveals that below 263 K, ion migration is suppressed, and the conductivity in this low-temperature regime is predominantly governed by electronic transport. Once the temperature exceeds 263 K, a sharp increase in conductivity is observed, indicating the onset of ion migration within the perovskite film. The activation energy (*Ea*) of ion migration is estimated according to the Nernst–Einstein relation:

2 $${\sigma}_{(T)}\mathrm{T}={\sigma}_{0}\mathrm{e}\mathrm{x}\mathrm{p}\left(-\frac{{E}_{a}}{k\mathrm{T}}\right)$$

where *σ*_0_ is a constant, *k* is the Boltzmann’s constant, and T is the temperature [68]. For the control sample, the *Ea* is 170 meV, whereas it increases to 220 meV in the GVL-based sample. This enhancement suggests that ion migration is effectively suppressed in the GVL-based films due to a reduced defect density. This result is further corroborated by the time-dependent PL spectra as shown in Fig. 4d, e, which directly reflect the phase segregation process. After 34 min of continuous illumination, the PL peak of the control film red-shifts by 6 nm, indicating the formation of an iodine-rich phase. In contrast, the PL peak position of the GVL-based film remains unchanged, suggesting superior photoresistance, which is consistent with the enhanced photostability observed in GVL-based PSCs [69]. We further employ conductive atomic force microscopy (c-AFM) with nanoscale resolution to differentiate the charge transport behavior within grain interiors and at grain boundaries. As per Fig. S21a, b, compared with control film, the perovskite film prepared by GVL exhibit brighter current mapping images, indicating that the latter possess superior electrical conductivity. Furthermore, the current line profiles obtained from the c-AFM images (Fig. S21c) reveal a uniform current distribution in the GVL-based film, while significant variations between grains and grain boundaries are observed in the control film. These results suggest that the GVL-based film exhibits lower conductivity at the grain boundaries, indicating fewer defects in these regions, which can suppress charge recombination and reduce dark current [70].

From the perspective of intact devices, we performed light intensity (I)-dependent *V*_OC_ measurements to further investigate the charge extraction and recombination behaviors. Figure 4f illustrates a linear correlation between *V*_OC_ and ln(I), and the GVL-based device exhibits a lower ideality factor compared to the control, indicating that trap-assisted Shockley–Read–Hall SRH recombination is effectively suppressed. This finding aligns well with the observed improvements in *V*_OC_ and FF [71]. The transient photovoltage (TPV) measurements depicted in Fig. 4g reveal that the GVL-based device exhibits a longer carrier recombination lifetime, which is primarily ascribed to the suppressed recombination dynamics [72]. The Mott–Schottky plots as shown in Fig. 4h indicate that the GVL-based devices exhibit a built-in potential (*V*_bi_) of 1.03 V, outperforming the control device of 0.91 V. This finding suggests that the GVL-based devices possess a higher driving force to enhance charge segregation and result in a small *V*_OC_ deficit [73]. Subsequently, we carried out thermal admittance spectroscopy (TAS) analysis to further explore the influence of GVL incorporation on the energy level distributions and the density of trap states [74]. As shown in Fig. 4i, compared with the control device, the GVL-based device exhibits relatively lower trap densities (Nt) in the energy depth ranges of 0.35–0.5 eV and beyond 0.6 eV. Overall, these findings collectively demonstrate that GVL incorporation effectively mitigates defect-related issues, thereby enhancing carrier dynamics, suppressing ion migration and phase segregation, and ultimately improving both efficiency and operational stability of the devices [75].

## Conclusion

In summary, for the first time, we present the ML-assisted co-solvent screening strategy to identify an eco-friendly solvent, *γ*-valerolactone (GVL), capable of partially replacing DMF while effectively regulating the crystallization dynamics of WBG perovskite films. Through strong interactions with FA⁺, GVL modulates crystallization kinetics and improves film quality by enhancing crystallinity, refining morphology, promoting beneficial facet exposure, and increasing orientational uniformity. This co-solvent strategy effectively suppresses phase separation and non-radiative recombination losses, while also facilitating charge transport. As a result, GVL-based WBG PSCs achieve a PCE of 23.3%, which ranks among the best reported values for this class of devices. In addition, the devices exhibit excellent long-term storage and operational stability. The GVL-based approach is also applicable to large-area and micron-thick perovskite films, delivering high efficiency and reproducibility. Furthermore, monolithic perovskite/silicon tandem devices fabricated with this strategy reach a PCE of 32.5%. This work not only establishes a sustainable approach to solvent engineering but also underscores the broader value of ML in guiding rational solvent design for advanced photovoltaic technologies.

## Supplementary Information

Below is the link to the electronic supplementary material.

Supplementary file1 (DOCX 11455 kb)
