# Supplementary material for: Artificial Intelligence-Guided Cosolvent Design for High-Performance Perovskite/Silicon Tandem Solar Cells
Source: Nanomicro Lett. 2026 Jul 21;18:446. doi: 10.1007/s40820-026-02291-9 (PMC13388578; doi:10.1007/s40820-026-02291-9)
Supplement: Supplementary file 1 — Supplementary file1 (DOCX 11455 kb) [file 40820_2026_2291_MOESM1_ESM.docx]

Supporting Information for

**Artificial Intelligence-Guided Cosolvent Design for High-Performance Perovskite/Silicon Tandem Solar Cells**

Lu Liu^1, 2, #^, Xinying Cai^2, #^, Bita Farhadi^3^, Xinrui Dong^1^, Kai Wang^1,*^, Yufei Shao^1^, Shulin Wang^1^, Jiaxue You^2^, Wanyi Li^1^, Hao-Chung Kuo^4^, Hanying Wang^5^, Dong Yang^1,*^, Alex K.-Y. Jen^2,*^, Shengzhong (Frank) Liu^1,*^

^1^ State Key Laboratory of Photoelectric Conversion and Utilization of Solar Energy, Center of Materials Science and Optoelectronics Engineering, Dalian Institute of Chemical Physics, Chinese Academy of Sciences, Dalian 116023, P. R. China

^2^ Department of Materials Science and Engineering, City University of Hong Kong, Kowloon 999077, Hong Kong, P. R. China

^3^ EIT Data Science and Communication College, Zhejiang Yuexiu University, Shaoxing 312030, P. R. China

^4^ Semiconductor Research Center, Hon Hai Research Institute, Taipei, Taiwan, P. R. China

^5^ School of New Energy, Yulin University, Yulin 719000, P. R. China

^#^ Lu Liu and Xinying Cai contributed equally to this work.

*Corresponding authors. E-mail: wangkai@dicp.ac.cn (Kai Wang); dongyang@dicp.ac.cn (Dong Yang); alexjen@cityu.edu.hk (Alex K.-Y. Jen); szliu@dicp.ac.cn (Shengzhong (Frank) Liu)

**S1 Supporting Figures**

**Fig. S1** SEM images of the (a) control; (b) 10 vol% GVL; (c) 20 vol% GVL; (c) 30 vol% GVL perovskite films. The scale bar is 1 μm. Inserts are statistical grain sizes.

**Fig. S2** (a) Digital photos of peeling off perovskite films from substrates with an epoxy encapsulant. Above and below are of the control and GVL-based films and substrates after peeling, respectively. Top-view SEM images of perovskite-substrate interface (b) Control and (c) GVL. The scale bar is 1 μm.

**Fig. S3** The XRD patterns of perovskite films prepared by different volume ratio of GVL.

**Fig. S4** Calculated adsorption energy of the (100), (110) and (111) crystal facets adsorbed by GVL.

**Fig. S5** The statistics of (a) PCE; (b) *V*_OC_; (c) *J*_SC_; (d) FF of control and PSCs based on different ratio of volume from 15 devices, respectively.

**Fig. S6** A summary of previous reported (a) PCE; (b) *V*_OC_ × FF of 1.67 eV wide-bandgap PSCs and comparation with the champion device in this work. The detailed parameters and other information are summarized in Table S4.

**Fig. S7** Stabilized PCE of control and GVL PSCs.

**Fig. S8** Champion *J−V* curves of control and GVL PSCs with active-area of 1.0 cm^2^.

**Fig. S9** A summary of previous reported PCE of wide-bandgap PSCs with active-area of ~ 1 cm^2^ and comparation with the champion device in this work. The detailed parameters and other information are summarized in Table S5.

**Fig. S10** Stabilized PCE and current density of control and GVL PSCs of PSCs with active-area of 1.0 cm^2^.

**Fig. S11** Statistical results of photovoltaic parameters including (a) PCE; (b) *V*_OC_; (c) *J*_SC_; (d) FF of control and GVL devices (1 cm^2^).

**Fig. S12** Cross-sectional SEM of perovskite film based on 1.8 M precursor solution.

**Fig. S13** Champion *J−V* curves of control and GVL PSCs with 1.8 M.

**Fig. S14** Histograms of the PCEs from 18 devices.

**Fig. S15** (a) Top-view, (b) cross-sectional SEM images of textured silicon bottom cell.

**Fig. S16** Stabilized power output of the champion TSC.

**Fig. S17** PCE distributions of 16 individual tandem devices.

**Fig. S18** Evolution of parameters for shelf stability of unencapsulated devices at 10% RH: (a) *V*_OC_, (b) *J*_SC_, (c) FF. Six devices for each type.

**Fig. S19** The water contact angles of (a) control; (b) GVL perovskite films.

**Fig. S20** XRD pattern of the control and GVL perovskite films after aging in ambient atmosphere for 13 days. # δ-CsPbI_3_, & δ-FAPbI_3_, * PbI_2_.

**Fig. S21** c-AFM images of (a) control, (b) GVL perovskite films. The scale bar is 1 μm; (c) Current along scan distance. The scan is along a line shown in the picture of (a) and (b).

**S2 Supporting Tables**

**Table S1** Detailed definitions of the 13 features screened as input X in machine learning model

| Feature | Detailed definition | Feature | Detailed definition |
| --- | --- | --- | --- |
| logP | Lipid-water partition coefficient used to describe hydrophobicity | HBD | Hydrogen bond donor count |
| Area | Measured area of the perovskite | HBA | Hydrogen bond acceptor count |
| TPSA | Topological polar surface area | RB | Rotatable bond count |
| MW | Molar volume | C | Number of carbon atom |
| O | Number of oxygen atom | N | Number of nitrogen atom |
| S | Number of sulfur atom | P | Number of phosphorus atom |
| PCE | Power conversion efficiency |  |  |

**Table S2** The donor number (D_N_) of related solvent

| Solvent | D_N_ (kcal/mol) |
| --- | --- |
| DMF | 26.6 |
| DMSO | 29.8 |
| GVL | 18.1 |
| THF | 20 |
| 2-MeTHF | 21 |
| 1-P | 15 |

**Table S3** *J-V* parameters and hysteresis index (HI) of the champion devices with and without GVL

| Sample | Scan direction | *V*_OC_ (V) | *J*_SC_  (mA cm^−2^) | FF (%) | PCE (%) | HI (%) |
| --- | --- | --- | --- | --- | --- | --- |
| Control | Reverse | 1.210 | 21.34 | 84.34 | 21.79 | 14.6 |
|  | Forward | 1.226 | 21.45 | 70.82 | 18.60 |  |
| GVL | Reverse | 1.257 | 21.64 | 85.63 | 23.30 | 6.1 |
|  | Forward | 1.249 | 21.61 | 81.09 | 21.89 |  |

**Table S4** Summary of the cosolvent systems applied in WBG PSCs

| Bandgap (eV) | Solvent | PCE (%) | Ref. |
| --- | --- | --- | --- |
| 1.68 | DMSO/ACN/EtOH | 21.5 | [S34] |
| 1.78 | DMSO/ACN/EtOH | 19.6 |  |
| 1.65 | DMF/DMSO/DMPU | 21.9 | [S35] |
| 1.72 | DMF/NMP | 19.46 | [S36] |
| 1.6 | DMF/DMSO/EtOH | 22.5 | [S37] |
| 1.68 | ACN/MA(MeOH) | 18.05 | [S38] |
| 1.75 | DMF/DMSO/formamide | 17.8 | [S39] |
| 1.76 | DMF/DMSO/DMI | 21.42 | [S40] |
| 1.67 | DMF/DMSO/GVL | 23.30 | This work |

**Table S5** Summary of photovoltaic parameters of reported WBG PSCs with bandgap of 1.67 eV in recent years

| *V*_OC_ (V) | *J*_SC_ (mA/cm^2^) | FF (%) | PCE (%) | *V*_OC_ × FF | Ref. |
| --- | --- | --- | --- | --- | --- |
| 1.225 | 19.49 | 79 | 18.94 | 0.97 | [S1] |
| 1.185 | 20.9 | 80.7 | 20.0 | 0.96 | [S2] |
| 1.195 | 21.10 | 80.88 | 20.64 | 0.97 | [S3] |
| 1.19 | 20.33 | 81.69 | 19.76 | 0.97 | [S4] |
| 1.200 | 20.67 | 77.0 | 19.09 | 0.92 | [S5] |
| 1.26 | 20.5 | 82.6 | 21.3 | 1.04 | [S6] |
| 1.14 | 22.06 | 80.88 | 20.34 | 0.92 | [S7] |
| 1.24 | 21.18 | 83.7 | 21.82 | 1.04 | [S8] |
| 1.26 | 20.31 | 79.81 | 20.17 | 1.01 | [S9] |
| 1.19 | 21.56 | 79.86 | 20.57 | 0.95 | [S10] |
| 1.21 | 21.3 | 83.3 | 21.47 | 1.01 | [S11] |
| 1.22 | 22.67 | 80.35 | 22.28 | 0.98 | [S12] |
| 1.29 | 22.32 | 85 | 24.48 | 1.10 | [S13] |
| 1.19 | 21.25 | 82.12 | 21.3 | 0.98 | [S14] |
| 1.26 | 20.05 | 81.57 | 20.61 | 1.03 | [S15] |
| 1.24 | 21.3 | 82.7 | 21.9 | 1.03 | [S16] |
| 1.25 | 21.05 | 84.34 | 22.06 | 1.05 | [S17] |
| 1.24 | 21.7 | 85.5 | 23.1 | 1.06 | [S18] |
| 1.219 | 21.08 | 81.1 | 20.85 | 0.99 | [S19] |
| 1.262 | 20.9 | 82.7 | 21.8 | 1.04 | [S20] |
| 1.246 | 21.49 | 86.53 | 23.18 | 1.08 | [S21] |
| 1.272 | 21.09 | 82.01 | 22 | 1.04 | [S22] |
| 1.24 | 22.02 | 84.34 | 23.02 | 1.05 | [S23] |
| 1.249 | 21.62 | 83.98 | 22.68 | 1.05 | [S24] |
| 1.246 | 21.68 | 85.34 | 23.05 | 1.06 | [S25] |
| 1.29 | 20.4 | 81.3 | 21.4 | 1.05 | [S26] |
| 1.23 | 21.43 | 81.36 | 21.42 | 1.00 | [S27] |
| 1.242 | 21.26 | 82.57 | 21.8 | 1.03 | [S28] |
| 1.28 | 21.87 | 83.06 | 23.25 | 1.06 | [S29] |
| 1.267 | 21.52 | 85.1 | 23.3 | 1.08 | [S30] |
| 1.295 | 21.60 | 84.95 | 23.46 | 1.10 | [S31] |
| 1.263 | 22.15 | 84.39 | 23.60 | 1.07 | [S32] |
| 1.268 | 21.7 | 86.8 | 23.42 | 1.10 | [S33] |
| 1.257 | 21.64 | 85.63 | 23.30 | 1.08 | This work |

**Table S6** Summary of photovoltaic parameters of reported WBG PSCs with area of ~1 cm^2^ in recent years

| Bandgap (eV) | *V*_OC_ (V) | *J*_SC_ (mA/cm^2^) | FF (%) | PCE (%) | Ref. |
| --- | --- | --- | --- | --- | --- |
| 1.85 | 1.396 | 16.25 | 67.93 | 15.41 | [S41] |
| 1.80 | 1.28 | 16.4 | 80.4 | 16.8 | [S42] |
| 1.79 | 1.309 | 17.61 | 80.4 | 18.54 | [S43] |
| 1.78 | 1.31 | 18.3 | 82.3 | 19.6 | [S44] |
| 1.77 | 1.285 | 18.32 | 79.48 | 18.71 | [S45] |
| 1.77 | 1.25 | 18.04 | 79.92 | 18.08 | [S46] |
| 1.77 | 1.31 | 17.80 | 79.18 | 18.46 | [S47] |
| 1.68 | 1.24 | 20.6 | 84.0 | 21.5 | [S44] |
| 1.68 | 1.272 | 20.74 | 83.98 | 22.16 | [S48] |
| 1.68 | 1.24 | 21.76 | 84.67 | 22.87 | [S49] |
| 1.67 | 1.272 | 20.53 | 80.48 | 20.54 | [S22] |
| 1.67 | 1.26 | 20.15 | 76.98 | 19.63 | [S17] |
| 1.67 | 1.23 | 21.79 | 81.58 | 21.86 | [S29] |
| 1.68 | 1.29 | 21.07 | 83.48 | 22.77 | [S50] |
| 1.67 | 1.215 | 21.63 | 80.37 | 21.11 | This work |

**Table S7** The detailed fitting parameters of TRPL results

| Sample | τ_ave_ (ns) | τ_1_ (ns) | τ_2_ (ns) | A_1_ | A_2_ |
| --- | --- | --- | --- | --- | --- |
| Control | 128.92 | 6.51 | 134.93 | 432.98 | 424.85 |
| GVL | 182.51 | 4.47 | 184.62 | 300.72 | 613.63 |

**Supplementary References**

1. F. Hou, Y. Li, L. Yan, B. Shi, N. Ren et al., Control perovskite crystals vertical growth for obtaining high-performance monolithic perovskite/silicon heterojunction tandem solar cells with *V*_OC_ of 1.93 V. Sol. RRL **5**(10), 2100357 (2021). <https://doi.org/10.1002/solr.202100357>
2. J. Liang, C. Chen, X. Hu, M. Xiao, C. Wang et al., Revealing the mechanism of π aromatic molecule as an effective passivator and stabilizer in highly efficient wide-bandgap perovskite solar cells. Sol. RRL **5**(8), 2100249 (2021). <https://doi.org/10.1002/solr.202100249>
3. J. Tao, X. Liu, J. Shen, S. Han, L. Guan et al., F-type pseudo-halide anions for high-efficiency and stable wide-band-gap inverted perovskite solar cells with fill factor exceeding 84. ACS Nano **16**(7), 10798–10810 (2022). <https://doi.org/10.1021/acsnano.2c02876>
4. R. Li, B. Chen, N. Ren, P. Wang, B. Shi et al., CsPbCl_3_-cluster-widened bandgap and inhibited phase segregation in a wide-bandgap perovskite and its application to NiO*_x_*-based perovskite/silicon tandem solar cells. Adv. Mater. **34**(27), 2201451 (2022). <https://doi.org/10.1002/adma.202201451>
5. W. Yang, H. Long, X. Sha, J. Sun, Y. Zhao et al., Unlocking voltage potentials of mixed-halide perovskite solar cells *via* phase segregation suppression. Adv. Funct. Mater. **32**(12), 2110698 (2022). <https://doi.org/10.1002/adfm.202110698>
6. G. Wang, J. Zheng, W. Duan, J. Yang, M.A. Mahmud et al., Molecular engineering of hole-selective layer for high band gap perovskites for highly efficient and stable perovskite-silicon tandem solar cells. Joule **7**(11), 2583–2594 (2023). <https://doi.org/10.1016/j.joule.2023.09.007>
7. A.Z. Afshord, B.E. Uzuner, W. Soltanpoor, S.H. Sedani, T. Aernouts et al., Efficient and stable inverted wide-bandgap perovskite solar cells and modules enabled by hybrid evaporation-solution method. Adv. Funct. Mater. **33**(31), 2301695 (2023). <https://doi.org/10.1002/adfm.202301695>
8. Y. Yang, Q. Chang, Y. Yang, Y. Jiang, Z. Dai et al., Multifunctional molecule interface modification for high-performance inverted wide-bandgap perovskite cells and modules. J. Mater. Chem. A **11**(31), 16871–16877 (2023). <https://doi.org/10.1039/D3TA02209A>
9. X. Zhang, X. Li, L. Tao, Z. Zhang, H. Ling et al., Precise control of crystallization and phase-transition with green anti-solvent in wide-bandgap perovskite solar cells with open-circuit voltage exceeding 1.25 V. Small **19**(22), 2208289 (2023). <https://doi.org/10.1002/smll.202208289>
10. X. Liu, J. Zhang, L. Tang, J. Gong, W. Li et al., Over 28% efficiency perovskite/Cu(*InGa*)Se_2_ tandem solar cells: highly efficient sub-cells and their bandgap matching. Energy Environ. Sci. **16**(11), 5029–5042 (2023). <https://doi.org/10.1039/D3EE00869J>
11. R. Wang, M. Li, Z. Ma, Z. He, Y. Dong et al., Hexachlorotriphosphazene-assisted buried interface passivation for stable and efficient wide-bandgap perovskite solar cells. Chem. Commun. **59**(41), 6255–6258 (2023). <https://doi.org/10.1039/d3cc01100c>
12. P. Hang, C. Kan, B. Li, Y. Yao, Z. Hu et al., Highly efficient and stable wide-bandgap perovskite solar cells *via* strain management. Adv. Funct. Mater. **33**(11), 2214381 (2023). <https://doi.org/10.1002/adfm.202214381>
13. Z. Fang, B. Deng, Y. Jin, L. Yang, L. Chen et al., Surface reconstruction of wide-bandgap perovskites enables efficient perovskite/silicon tandem solar cells. Nat. Commun. **15**, 10554 (2024). <https://doi.org/10.1038/s41467-024-54925-4>
14. Z. Huang, X. Ge, Z. Liu, B. Shi, P. Wang et al., Highly efficient blade-coated 1.67 eV p-i-n perovskite solar cells enabled by a hybrid self-assembled monolayer and surface passivation. ACS Appl. Energy Mater. **7**(24), 11683–11690 (2024). https://doi.org/10.1021/acsaem.4c01080
15. X. Huo, S. Mariotti, Y. Li, T. Guo, C. Ding et al., Unraveling the relationship between the phenethylammonium-induced 2D phase on the perovskite surface and inverted wide bandgap perovskite solar cell performance. Energy Environ. Sci. **17**(22), 8658–8669 (2024). <https://doi.org/10.1039/d4ee02133a>
16. G. Wang, W. Duan, Q. Lian, M.A. Mahmud, T.L. Leung et al., Reducing voltage loss *via* dipole tuning for electron-transport in efficient and stable perovskite-silicon tandem solar cells. Adv. Energy Mater. **14**(40), 2401029 (2024). <https://doi.org/10.1002/aenm.202401029>
17. P. Jia, G. Chen, G. Li, J. Liang, H. Guan et al., Intermediate phase suppression with long chain diammonium alkane for high performance wide-bandgap and tandem perovskite solar cells. Adv. Mater. **36**(25), 2400105 (2024). <https://doi.org/10.1002/adma.202400105>
18. S. Li, Z. Zheng, J. Ju, S. Cheng, F. Chen et al., A generic strategy to stabilize wide bandgap perovskites for efficient tandem solar cells. Adv. Mater. **36**(9), 2307701 (2024). <https://doi.org/10.1002/adma.202307701>
19. Z.-W. Tao, T. Lu, X. Gao, M.U. Rothmann, Y. Jiang et al., Heterogeneity of light-induced open-circuit voltage loss in perovskite/Si tandem solar cells. ACS Energy Lett. **9**(4), 1455–1465 (2024). <https://doi.org/10.1021/acsenergylett.4c00110>
20. Z. Liu, H. Li, Z. Chu, R. Xia, J. Wen et al., Reducing perovskite/C_60_ interface losses *via* sequential interface engineering for efficient perovskite/silicon tandem solar cell. Adv. Mater. **36**(8), 2308370 (2024). <https://doi.org/10.1002/adma.202308370>
21. L. Liu, B. Farhadi, J. Li, S. Liu, L. Lu et al., Hydrophobic hydrogen-bonded polymer network for efficient and stable perovskite/Si tandem solar cells. Angew. Chem. Int. Ed. **63**(8), e202317972 (2024). <https://doi.org/10.1002/anie.202317972>
22. L. Qiao, T. Ye, T. Wang, W. Kong, R. Sun et al., Freezing halide segregation under intense light for photostable perovskite/silicon tandem solar cells. Adv. Energy Mater. **14**(7), 2302983 (2024). <https://doi.org/10.1002/aenm.202302983>
23. Y. Zhang, N. University, N. University, X. Dong et al., Multifunctional phenothiazine-based self-assembled monolayer as a hole-selective contact for efficient wide-band-gap perovskite solar cells. Nano Lett. **25**(29), 11257–11265 (2025). <https://doi.org/10.1021/acs.nanolett.5c02152>
24. Z. Luo, L. Tang, L. Zeng, H. Fang, W. Wang et al., Albendazole passivation in inverted wide-bandgap perovskite solar cells toward efficient perovskite/CuInGaSe(2) tandem photovoltaics. Adv. Mater. **37**(41), e05597 (2025). <https://doi.org/10.1002/adma.202505597>
25. S. Wu, M. Hu, J. Wang, J. Zhu, W. Jiao et al., Customized multifunctional additive regulates 1.67 eV-wide-bandgap perovskite crystallization for four-terminal perovskite/silicon tandem solar cells. Adv. Mater. **37**(26), 2503269 (2025). <https://doi.org/10.1002/adma.202503269>
26. L. Zheng, M. Wei, F.T. Eickemeyer, J. Gao, B. Huang et al., Strain-induced rubidium incorporation into wide-bandgap perovskites reduces photovoltage loss. Science **388**(6742), 88–95 (2025). <https://doi.org/10.1126/science.adt3417>
27. J. Kim, J. Park, G. Kim, W. Xu, S.D. Stranks et al., Quasi-2D scaffolding for enhanced stability and efficiency in 1.67 eV Cs-rich pure-iodide perovskite solar cells. Small **21**(16), 2500197 (2025). <https://doi.org/10.1002/smll.202500197>
28. W. Li, X. Liu, J. Zhang, H. Wang, C. Yuan et al., Reactive plasma deposition of ITO as an efficient buffer layer for inverted perovskite solar cells. Adv. Mater. **37**(12), 2417094 (2025). <https://doi.org/10.1002/adma.202417094>
29. Y. Yang, Q. Chang, J. Su, L. Chao, Y. Wang et al., Activating halogen circulation enables efficient and stable wide-bandgap mixed-halide perovskite solar cells. Adv. Mater. **37**(11), 2416513 (2025). <https://doi.org/10.1002/adma.202416513>
30. L. Tian, E. Bi, I. Yavuz, C. Deger, Y. Tian et al., Divalent cation replacement strategy stabilizes wide-bandgap perovskite for Cu(In, Ga)Se_2_ tandem solar cells. Nat. Photonics **19**(5), 479–485 (2025). <https://doi.org/10.1038/s41566-025-01618-z>
31. X. Zhang, S. Wang, Z. Zhang, L. Ma, L. Tao et al., Minimizing the V OC deficit by regulating facets of wide-bandgap perovskite for monolithic perovskite/silicon tandem solar cells. eScience **6**(4), 100563 (2026). <https://doi.org/10.1016/j.esci.2026.100563>
32. S. Wu, Z. Wu, J. Li, L. Yu, Z. Song et al., Potassium cyanate stabilizes lattice and promotes preferred orientation for 1.67-eV wide-bandgap perovskite and perovskite/silicon tandems. Adv. Mater. **38**(11), e19976 (2026). <https://doi.org/10.1002/adma.202519976>
33. L.-C. Chang, T. Duong, V. Ahmad, H. Zhan, A.D. Bui et al., High-efficiency perovskite/silicon tandem solar cells based on wide-bandgap perovskite solar cells with unprecedented fill factor. Nano-Micro Lett. **18**(1), 122 (2026). <https://doi.org/10.1007/s40820-025-01959-y>
34. C. Duan, H. Gao, K. Xiao, V. Yeddu, B. Wang et al., Scalable fabrication of wide-bandgap perovskites using green solvents for tandem solar cells. Nat. Energy **10**(3), 318–328 (2025). <https://doi.org/10.1038/s41560-024-01672-x>
35. R. Wang, J. Zhu, J. You, H. Huang, Y. Yang et al., Custom-tailored solvent engineering for efficient wide-bandgap perovskite solar cells with a wide processing window and low VOC losses. Energy Environ. Sci. **17**(7), 2662–2669 (2024). <https://doi.org/10.1039/D4EE00330F>
36. C. Tian, X. Gao, J. Li, J. Pan, G. Yu et al., Scalable growth of stable wide-bandgap perovskite towards large-scale tandem photovoltaics. Sol. RRL **6**(7), 2200134 (2022). <https://doi.org/10.1002/solr.202200134>
37. Q. Liang, K. Liu, M. Sun, Z. Ren, P.W.K. Fong et al., Manipulating crystallization kinetics in high-performance blade-coated perovskite solar cells *via* cosolvent-assisted phase transition. Adv. Mater. **34**(16), 2200276 (2022). <https://doi.org/10.1002/adma.202200276>
38. A.S. Subbiah, F.H. Isikgor, C.T. Howells, M. De Bastiani, J. Liu et al., High-performance perovskite single-junction and textured perovskite/silicon tandem solar cells *via* slot-die-coating. ACS Energy Lett. **5**(9), 3034–3040 (2020). <https://doi.org/10.1021/acsenergylett.0c01297>
39. J. Kim, M.I. Saidaminov, H. Tan, Y. Zhao, Y. Kim et al., Amide-catalyzed phase-selective crystallization reduces defect density in wide-bandgap perovskites. Adv. Mater. **30**(13), 1706275 (2018). <https://doi.org/10.1002/adma.201706275>
40. Y. Li, X. Zhao, N. Meng, S. Dong, S. Yan et al., Synchronous phase transformation for efficient wide-bandgap perovskite photovoltaics. Adv. Mater. **37**(40), e05694 (2025). <https://doi.org/10.1002/adma.202505694>
41. G. Xie, H. Li, J. Fang, X. Wang, H. Peng et al., Crystallization thermodynamics regulation of 1.85 eV wide-bandgap perovskite for efficient and stable perovskite-organic tandem photovoltaics. Angew. Chem. Int. Ed. **64**(17), e202501764 (2025). <https://doi.org/10.1002/anie.202501764>
42. P. Caprioglio, J.A. Smith, R.D.J. Oliver, A. Dasgupta, S. Choudhary et al., Open-circuit and short-circuit loss management in wide-gap perovskite p-i-n solar cells. Nat. Commun. **14**, 932 (2023). <https://doi.org/10.1038/s41467-023-36141-8>
43. L. Qiao, T. Ye, P. Wang, T. Wang, L. Zhang et al., Crystallization enhancement and ionic defect passivation in wide-bandgap perovskite for efficient and stable all-perovskite tandem solar cells. Adv. Funct. Mater. **34**(7), 2308908 (2024). <https://doi.org/10.1002/adfm.202308908>
44. C. Duan, H. Gao, K. Xiao, V. Yeddu, B. Wang et al., Scalable fabrication of wide-bandgap perovskites using green solvents for tandem solar cells. Nat. Energy **10**(3), 318–328 (2025). <https://doi.org/10.1038/s41560-024-01672-x>
45. D. Pu, S. Zhou, H. Guan, P. Jia, G. Chen et al., Enhancing efficiency and intrinsic stability of large-area blade-coated wide-bandgap perovskite solar cells through strain release. Adv. Funct. Mater. **34**(17), 2314349 (2024). <https://doi.org/10.1002/adfm.202314349>
46. A. Zhang, M. Li, C. Dong, W. Ye, X. Yang et al., π–π stacking at the perovskite/C60 interface enables high-efficiency wide-bandgap perovskite solar cells. Small **20**(35), 2401197 (2024). <https://doi.org/10.1002/smll.202401197>
47. R. He, W. Wang, Z. Yi, F. Lang, C. Chen et al., Improving interface quality for 1-Cm2 all-perovskite tandem solar cells. Nature **618**(7963), 80–86 (2023). <https://doi.org/10.1038/s41586-023-05992-y>
48. T. Ye, L. Qiao, T. Wang, P. Wang, L. Zhang et al., Molecular bridge in wide-bandgap perovskites for efficient and stable perovskite/silicon tandem solar cells. Adv. Funct. Mater. **35**(25), 2419391 (2025). <https://doi.org/10.1002/adfm.202419391>
49. Y. Yao, B. Li, D. Ding, C. Kan, P. Hang et al., Oriented wide-bandgap perovskites for monolithic silicon-based tandems with over 1000 hours operational stability. Nat. Commun. **16**, 40 (2025). <https://doi.org/10.1038/s41467-024-55377-6>
50. Z. Han, Z. Wang, Z. Xia, X. Zhang, J. Yang et al., Uniform phase distribution of wide bandgap perovskite for high-performance perovskite-silicon tandem solar cells. Nat. Commun. **16**, 11470 (2025). <https://doi.org/10.1038/s41467-025-66480-7>
